# Performance Evaluation of the Luminex Aries C. difficile Assay in Comparison to Two Other Molecular Assays within a Multihospital Health Care Center

**DOI:** 10.1128/JCM.01092-19

**Published:** 2019-10-23

**Authors:** Stefan Juretschko, Ryhana Manji, Reeti Khare, Shubhagata Das, Sherry Dunbar

**Affiliations:** a Pathology and Laboratory Medicine, Northwell Health Laboratories, Little Neck Parkway, New York, USA; b Luminex Cooperation, Austin, Texas, USA; Medical College of Wisconsin

**Keywords:** *Clostridioides difficile*, *C. difficile* infection, molecular assays, Aries, Xpert, BD Max, *Clostridium difficile*, real-time PCR, toxins

## Abstract

Clostridioides difficile infection (CDI) remain a serious issue in the United States. Fast and accurate diagnosis of CDI is paramount to achieve immediate infection control initiation, triaging, and isolation, as well as appropriate antibiotic treatment. However, both, over- and underdiagnosis can lead to adverse patient outcomes, such as unnecessary administration of antibiotics or unwanted spread of spores in any hospital setting, respectively. In this prospective study, we evaluated the FDA-cleared Aries C. difficile assay and compared its performance and workflow characteristics to those of the BD Max Cdiff and Xpert C. difficile/*Epi* assays. Out of 302 samples tested, 55 (18.2%) samples were positive, and 234 (77.5%) samples were negative for C. difficile by all three testing methods. Comparison results showed a positive and negative percent agreement (PPA and NPA, respectively) between the Aries and Xpert assays of 95.2% (59/62) and 99.2% (238/240), respectively. The PPA and NPA between the Aries and BD Max assays were 91.8% (56/61) and 96.6% (230/238), respectively. Invalid result rates were determined to be 2.6% for the BD Max assay, 1.0% for the Aries assay, and 0% for the Xpert assay. Hands-on time (HoT) and total turnaround time (TAT) varied considerably depending on the sample number and instrument throughput. The HoT ranged from 1.2 to 3.5 min per sample, and the TAT was 1 to 2.3 h. Overall, the results demonstrated that the Aries assay is a rapid and sensitive method for the diagnosis of CDI in clinical laboratories.

## Abstract (precis)

Clostridioides difficile infection (CDI) remain a serious issue in the United States. Fast and accurate diagnosis of CDI is paramount to achieve immediate infection control initiation, triaging, and isolation, as well as appropriate antibiotic treatment. However, both, over- and underdiagnosis can lead to adverse patient outcomes, such as unnecessary administration of antibiotics or unwanted spread of spores in any hospital setting, respectively.

## INTRODUCTION

Clostridioides difficile is an anaerobic, fastidious, spore-forming, Gram-positive bacillus that causes hospital-acquired diarrhea and antibiotic-associated pseudomembranous colitis. The bacterium produces harmful exotoxins, toxin A (TcdA) and toxin B (TcdB), that damage the colonic mucosa and are associated with a more severe form of the disease. According to the Centers for Disease Control and Prevention (CDC), C. difficile infection (CDI) is one of the most common hospital-acquired infections and is responsible for approximately 15,000 deaths annually in the United States. (https://www.cdc.gov/hai/organisms/cdiff/cdiff_infect.html). It has been estimated that CDI-associated hospital stays have increased by 9.2% between 2011 and 2015, and the average cost per case for hospital-onset CDI is about $34,157, which is 1.5 times the cost of community-onset CDI (1, 2). The clinical signs and symptoms presented by CDI are highly nonspecific, such as mild to moderate diarrhea, nausea, and abdominal cramps, and therefore, it is difficult to distinguish between patients with and without CDI (3). Several testing methods are currently available for the diagnosis of CDI, such as stool and toxigenic culture, cell cytotoxicity neutralization assays (CCNAs), glutamate dehydrogenase (GDH) assays, toxin detection by enzyme immunoassays (EIAs), and toxin gene detection by nucleic acid amplification tests (NAATs).

NAATs for the detection of C. difficile have rapidly evolved over the years, and currently, more than a dozen U.S. Food and Drug Administration (FDA)-approved NAATs are commercially available (https://www.fda.gov/medical-devices/vitro-diagnostics/nucleic-acid-based-tests). Most of these assays detect both the *tcdA* and *tcdB* genes for their corresponding toxins or the *tcdB* gene for TcdB only. Recent investigations show that TcdB is a more virulent toxin than TcdA in several cell types; however, the detection of both *tcdA* and *tcdB* may be important, as the individual contributions of the toxins in CDI pathogenesis remain ambiguous (4, 5).

The Aries C. difficile assay (Luminex Corporation, Austin, TX) is an automated NAAT for the qualitative detection of the *tcdA* and *tcdB* genes from stool samples from patients suspected of having CDI. The assay uses Luminex Corporation’s MultiCode chemistry in combination with the Luminex Aries system, an automated platform that performs nucleic acid extraction and purification, real-time PCR for the detection of target-specific DNA, and data analysis. The aim of this study was to evaluate the performance characteristics and workflow of the Aries C. difficile assay compared to those of the Xpert C. difficile/*Epi* (Cepheid, Sunnyvale, CA) and the BD Max Cdiff (Becton, Dickinson, Franklin Lakes, NJ) assays using deidentified, remnant stool samples.

(This study was presented in part at the 2018 American Society of Microbiology Annual Meeting in Atlanta, GA.)

## MATERIALS AND METHODS

### Samples.

A total of 302 selected deidentified remnant stool samples in Cary-Blair medium submitted for routine clinical testing by the Xpert assay were included in the study. Samples tested by the Xpert assay were deidentified and renumbered to mask the identity of the subject to the operators and investigator. The comparator method’s result prior to study testing was not known to the operators.

### Study design.

This prospective comparison study was performed at Northwell Health Laboratories (Lake Success, NY, USA) under an institutional review board (IRB)-approved protocol (Feinstein Institutes for Medical Research; no. 17-08-269-03). Deidentified remnant stool samples submitted for routine clinical testing by the Xpert assay were collected between 1 July and 15 October 2017. The samples were subsequently tested by the Aries and the BD Max assays. Any disagreement between all three NAATs was further investigated by toxin gene sequencing.

### Molecular testing.

All assays were performed according to the individual manufacturer’s instructions. For the Aries assay, unpreserved, raw stool samples were preprocessed using the Aries stool resuspension kit. Eight hundred microliters of Aries stool resuspension buffer was added to a 2-ml Aries stool resuspension tube. The primary stool sample (∼160 μl) was added using the Aries stool resuspension swab. The tube was vortexed for 15 s and centrifuged at 2,000 × *g* for 30 s. A total of 200 μl of the preprocessed stool was pipetted into the sample chamber of the Aries C. difficile assay cassette. The cassette was then placed into an Aries magazine, which was inserted into an Aries system. Internal scanning automatically associates the preloaded Aries C. difficile assay program with the cassette. Once the run is initiated, nucleic acid from the sample is extracted, purified, and amplified automatically within the Aries system and the C. difficile assay cassette. The total assay time, including extraction and PCR cycling, is approximately 2 h. Following completion, the results were reported as either toxigenic C. difficile positive, negative, or invalid. None of the samples underwent a freeze-thaw cycle, and all samples remaining after testing were frozen at less than –70°C for discordant analysis and/or confirmatory testing as needed.

### Discordant analysis.

All samples that showed disagreement with any of the three NAATs were further investigated using an alternative PCR with subsequent bidirectional sequencing. A 10-μl loopful (approximately 100 to 150 mg) of the stool sample was pretreated in 1 ml of easyMAG lysis buffer (bioMérieux, Inc., Durham, NC), 850 μl of the pretreated stool was extracted using the bioMérieux the NucliSENS easyMAG system, and 5 μl of the extracted nucleic acids was subjected to singleplex PCR. Bidirectional sequencing was performed using M13-tagged forward and reverse primers that targeted different regions of *tcdA* and *tcdB* than did the Aries C. difficile assay. Following PCR, unincorporated primers and deoxynucleoside triphosphates (dNTPs) were removed by treatment with exonuclease I and shrimp alkaline phosphatase (Thermo Fisher Scientific, Waltham, MA). The BigDye Terminator v3.1 cycle sequencing kit (Thermo Fisher) was used to perform dye-labeled terminator cycle sequencing with detection on the 3730xl analyzer (Thermo Fisher). BLAST analysis (NCBI) was conducted for sequences that were at least 200 bases in length, had a Phred score greater than or equal to 20 for at least 90% of the bases, and contained fewer than 5% ambiguous base calls. Sequences with greater than 95% query coverage and identity and an expected value (E value) of less than 10^−30^ compared to the reference sequence were considered positive. A weak-positive result may be obtained if the sequence that was generated was specific to the target but was only 100 to 199 nucleotides or was only detected in one direction.

### Time and motion analysis.

Workflow analysis of the Xpert, BD Max, and Aries assays was conducted on a single day. The workflow analysis was performed for different run sizes (one, six, and 12 samples) to mimic potential different work processes and volumes at other institutions. The following parameters were included: (i) hands-on time (HoT), including manual steps such as sample setup, initiation of the instrument, postrun tasks, and result reporting; and (ii) total turnaround time (TAT), including HoT and hands-off or automation time (AuT). The times required for the different phases of the assay were recorded by two observers independent of performing the assay. All assays were performed by experienced medical technologists who were familiar with the instruments and had prior training on the assay procedures.

### Statistical analysis.

The results were analyzed to determine the positive percent agreement (PPA) (no. of true positives/[no. of true positives + no. of false negatives]), negative percent agreement (NPA) (no. of true negatives/[no. of true negatives + no. of false positives]), and the overall percent agreement for Aries with each of the comparator platforms. True positivity/negativity was determined as two of three positives/negatives by any assay. Ninety-five percent confidence intervals (CIs), including continuity corrections, were calculated according to the procedure described by Newcombe (6) and outlined by Wilson (7).

## RESULTS

### Analytical performance.

Of the 302 samples included in this study, 55 (18.2%) samples were positive, and 234 (77.5%) samples were negative for C. difficile by all three assays. A total of 62 (20.5%) samples were positive by the Xpert assay, 61 (20.2%) samples were positive by the Aries assay, and 56 (18.5%) samples were positive by the BD Max assay (Table 1). The PPA, NPA, and overall agreement between the Aries and Xpert assays were 95.2% (59/62; 95% CI, 85.6% to 98.7%), 99.2% (238/240; 95% CI, 96.7% to 99.9%), and 98.3% (297/302), respectively (Table 2). The PPA, NPA, and overall agreement between the Aries and BD Max assays were 98.2% (55/56; 95% CI, 89.2% to 99.9%), 97.5% (236/242; 95% CI, 94.4% to 98.9%), and 97.7% (291/298), respectively (Table 3).

**TABLE 1:** Analysis of test results for all samples using the Aries, Xpert, and BD Max assays

Sample	Result by assay		
	Aries	Xpert	BD Max
55	+	+	+
4	+	+	−
2	+	−	−
2	−	+	−
1	−	+	+
3	−	−	Invalid^*a*^
1	−	−	Not determined^*b*^
234	−	−	−

a These specimens remained invalid after repeat testing.

b This specimen was invalid on initial testing and was not retested.

**TABLE 2:** Performance characteristics of the Aries assay compared to the Xpert assay

Aries result	No. with Xpert result of^*a*^:		Total no. of samples
	Positive	Negative	
Positive	59	2	61
Negative	3	238	241
Total	62	240	302

a The PPA and NPA were 95.2% (95% confidence interval [CI], 85.6% to 98.7%) and 99.2% (96.7% to 99.9%), respectively.

**TABLE 3:** Performance characteristics of the Aries assay compared to the BD Max assay

Aries assay result	No. with BD Max result of^*a*^:		Total no. of samples
	Positive	Negative	
Positive	55	6	61
Negative	1	236	237
Total	56	242	298^*b*^

a The PPA and NPA were 98.2% (95% CI, 89.2% to 99.9%) and 97.5% (94.4% to 98.9%), respectively.

b Four samples generated invalid results by the BD Max assay. Three of these samples remained invalid after repeat testing. One sample was not further tested. All four samples tested negative by the Aries and Xpert assays.

Comparison of the BD Max and Xpert assays revealed an overall agreement of 98.0% (292/298; 95% CI, 95.7% to 99.3%) and PPA and NPA of 90.3% (56/62; 95% CI, 79.5% to 96.0%) and 100.0% (236/236; 95% CI, 98.0% to 100.0%), respectively.

Eight (2.6%) samples yielded invalid results on the initial run by the BD Max assay. Seven of these samples were available for retesting, and four of them generated a valid negative result upon repeat testing. Three (1%) invalid results were obtained by the Aries assay; however, upon retesting, valid negative results were obtained. No samples yielded an invalid result with the Xpert assay.

### Discordant analysis.

A total of 13 (4.3%) samples yielded discrepant results where the three NAATs were not in agreement. One sample was not further tested and was eliminated from the discordant analysis. The dispositions of the 12 remaining discordant samples and analysis are shown in Table 4. Three samples tested negative by both the Aries and Xpert assays but produced an invalid result with BD Max assay even after retesting. Two of these samples were also negative by bidirectional sequencing, and one sample produced a weak-positive sequencing result for *tcdA*. A sequencing result was considered weak positive when the sequence generated was specific to the target but was only 100 to 199 nucleotides or was only detected in one direction. Two samples tested negative by both the Aries and BD Max assays but generated a positive result with the Xpert assay and bidirectional sequencing. One specimen tested positive by both the Xpert and BD Max assays but generated a negative result with the Aries assay (Aries false negative). This specimen was also positive by bidirectional sequencing. Two specimens were negative by both the Xpert and BD Max assays but generated a positive result for *tcdB* by the Aries assay. Upon sequencing, only one of these two specimens was confirmed as *tcdB* positive. Finally, four samples tested positive with the Aries and Xpert assays but generated negative results with the BD Max assay. Three of these samples were also positive by bidirectional sequencing.

**TABLE 4:** Results of discordant analysis for discrepant samples

Sample ID^*a*^	Result (gene[s]) by assay or method				Final call
	Aries	Xpert	BD Max	Bidirectional sequencing	
65	−	−	Invalid	−	Negative
158	−	−	Invalid	−	Negative
72	−	−	Invalid	+ (low positive, *tcdA)*	Negative
109	−	+	−	+ (*tcdA, tcdB*)	Positive
266	−	+	−	+ (*tcdA, tcdB*)	Positive
273	−	+	+	+ (*tcdA, tcdB*)	Positive
209	+ (*tcdB)*	−	−	−	Negative
243	+ (*tcdB)*	−	−	+ (*tcdB*)	Positive
226	+ (*tcdA, tcdB*)	+	−	−	Positive
82	+ (*tcdB*)	+	−	+ (*tcdA, tcdB*)	Positive
117	+ (*tcdB*)	+	−	+ (*tcdB*)	Positive
207	+ (*tcdA*)	+	−	+ (*tcdA, tcdB*)	Positive

a ID, identifier.

### Time and motion data.

**FIG 1:**
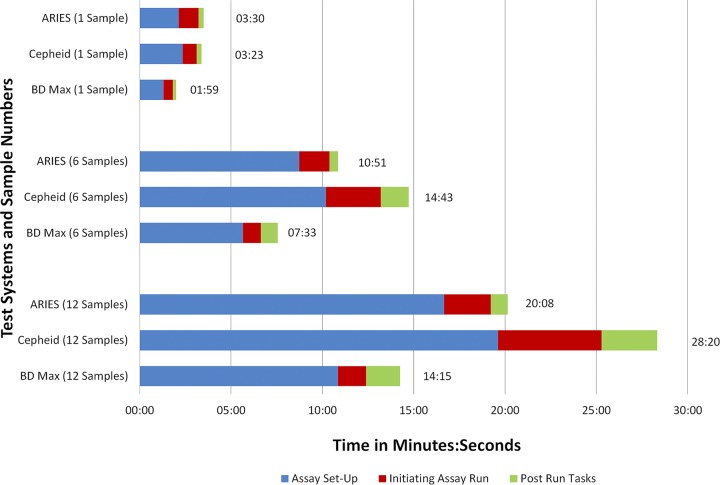
Comparison of hands-on times (HoT) for C. difficile test systems.

**FIG 2:**
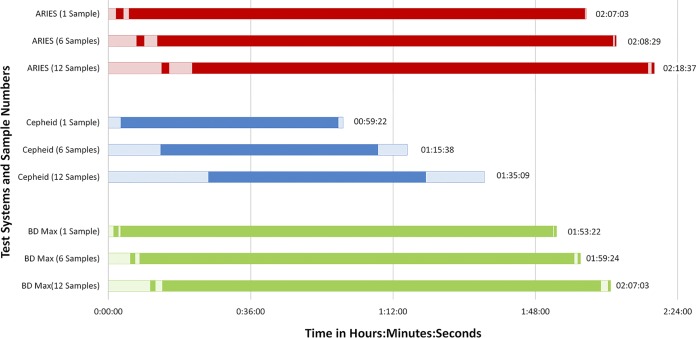
Comparison of total turnaround time (TAT) for C. difficile test systems. The hands-on time (HoT) is shown in light-shaded bars, and the automation time is shown in the dark-shaded bars.

The TAT and HoT varied depending on sample number and instrument throughput. The total HoT for testing one sample was less than 4 min (range, 0:01:59 to 0:03:30 h:min:s) for all three assays (Fig. 1). The HoT for testing one sample was lowest for the BD Max assay (0:01:59 h:min:s) and was highest for the Aries assay (0:03:30 h:min:s). However, as the number of samples increased to six and 12, the Xpert assay required the highest total HoT (0:14:43 and 0:28:20 h:min:s, respectively), whereas those for the Aries assay were 0:10:51 and 0:20:08 h:min:s, and the BD Max assay was fastest at 0:7:33 and 0:14:15 h:min:s, respectively. The total TAT for testing one, six, and 12 samples was highest for the Aries assay (2:00:51, 2:08:29, and 2:18:07 h:min:s, respectively) and lowest for the Xpert assay (0:59:22, 1:15:38, and 1:35:09 h:min:s, respectively), provided that sufficient bays are installed in the Xpert instrument (Fig. 2). The TAT for BD Max was in between, at 1:53:22, 1:59:24, and 2:07:03 h:min:s for testing one, six, and 12 samples, respectively.

## DISCUSSION

In this study, we compared the performance and workflow characteristics of three commercially available NAATs for C. difficile detection, the Aries C. difficile assay, the Xpert C. difficile/*Epi* assay, and the BD Max Cdiff assay. Three hundred two stool samples were collected from patients suspected of having CDI, and 55 (18.2%) samples were identified as positive for C. difficile by all three molecular assays. Analytical results demonstrated that all three molecular assays had comparable sensitivity and specificity (>91% PPA and >98% NPA) and were in agreement for 289 (95.7%) of the samples. All three molecular platforms had a relatively low invalid result rate (<3%), and these invalid rate data were in agreement with previous studies (8, 9).

Approximately 4% of the samples had discrepant results among the three molecular methods. One specimen tested negative by both the Aries and Xpert assays and was invalid on the BD Max assay but generated a positive result with bidirectional sequencing. However, the sequencing assay was weakly positive only for *tcdA* (1 of 2 replicates, 1 primer set only), indicating that the pathogen concentration might have been below the limit of detection for the Aries assay. The Xpert assay can only detect *tcdB* and therefore generated a negative result for this sample. Additionally, since the BD Max assay was repeatedly invalid for this specimen, sample inhibition may have also contributed to the discrepant results. Two samples tested positive by the Xpert assay but generated a negative result by the Aries and BD Max assays. Bidirectional sequencing for these samples was also positive. However, the sequencing assay uses more of the specimen than does the Aries assay, undergoes a separate off-board extraction, and is performed in singleplex, which may account for a higher sensitivity. Differences in the limits of detection (LODs) for the three assays might also be responsible for the discrepancies in the test results. According to the analytical performance data described in the U.S. FDA 510(k) summaries for these assays, the reported LODs of the Aries, BD Max, and Xpert assay are 5.10 CFU/cassette, 265 CFU/loop, and 460 CFU/swab, respectively (https://www.accessdata.fda.gov/). This suggests that the Aries is the most sensitive of the three assays, which could account for the Aries false-positive results. However, all three assays are highly sensitive and show very similar performance characteristics in this study.

Analysis of the time and motion data revealed that the BD Max assay has the lowest HoT for testing one sample, followed by the Xpert and Aries assays. As the number of samples increased to six and 12, the Xpert assay demonstrated the highest HoT, followed by the Aries and BD Max assays. The TAT was lowest for the Xpert assay, followed by the BD Max and Aries assays; however, TAT can differ considerably depending on the number of systems, modules, or bays installed for each platform. The Aries and BD Max systems include two bays or modules and can test 12 and 24 samples at a time, respectively. The Xpert system is available in 1-, 2-, 4-, 16-, 48-, or 80-module configurations, and therefore, the TAT could be considerable higher if fewer modules are installed than can accommodate the sample load. For the BD Max assay, the higher invalid result rate than those of the Aries and Xpert assays may further increase the TAT if repeat testing is considered in the TAT calculations.

The diagnostic paradigm for C. difficile has evolved and changed in recent years, but the optimal diagnostic method for CDI is still a controversial subject (10). Diagnosis and management of CDI are particularly challenging because of nonspecific clinical symptoms, high rates of asymptomatic colonization with both toxigenic and nontoxigenic strains of C. difficile, and the difficulties associated with identifying unstable exotoxin (11). According to the diagnostic guidelines published by the European Society of Clinical Microbiology and Infectious Diseases (ESCMID) and the Infectious Diseases Society of America (IDSA), effective diagnosis of CDI requires both clinical presentation of the symptoms and confirmatory laboratory evidence of C. difficile exotoxin- or toxin-producing C. difficile strains in stool samples (12, 13).

EIAs for toxins A and B are rapid and relatively inexpensive but have demonstrated poor sensitivity (47% to 72%) compared to molecular tests (14, 15). Additionally, no correlation has been observed between the active stool toxins measured by EIAs and disease severity in CDI (16). CCNAs and toxigenic cultures are more sensitive than EIAs; however, these assays are labor-intensive and time-consuming and can have a turnaround time of up to 3 days (17). GDH assays have demonstrated >90% sensitivity and specificity compared to culture and are commonly used as a screening test (18). However, since GDH is expressed by both toxigenic and nontoxigenic strains of C. difficile, confirmatory tests, such as NAATs or toxin assays, are required for positive results.

NAATs have demonstrated higher sensitivity than those of currently available toxin assays (92.2% to 97.7% versus 42.3% to 82.8%, respectively), and the more rapid diagnosis can positively impact patient care by decreasing empirical therapy among patients without CDI from 13.6% to 5.6% (19, 20). Additionally, since NAATs can detect more toxigenic C. difficile cases, implementation of these assays can facilitate improved infection control measures, thereby reducing the overall infection rate of the clinical institution (21, 22). Although most clinical laboratories have shifted from toxin assays to NAATs, the clinical utility of NAATs is still under debate for diagnosing CDI (10). Since NAATs target the toxin genes regardless of toxin production, a NAAT positive result does not always indicate clinical disease. Recent studies have demonstrated that relying exclusively on NAATs for the diagnosis of CDI can result in overdiagnosis, overtreatment, and an increase in health care costs (23, 24). To address this problem, current ESCMID guidelines recommend a multistep algorithm for diagnosing CDI that includes confirmation of a positive result by the first diagnostic test by one or two other confirmatory tests or reference methods (12). However, the latest guidelines from the IDSA recommend that NAATs be used as a standalone diagnostic test in cases where there are preagreed institutional criteria for patient stool submission (13). Thus, as there is a still lack of consensus regarding the best diagnostic method for CDI, clinical laboratories should develop and optimize their own diagnostic algorithm based on their patient population, CDI severity, and prevalence rate.

This study demonstrated that the Aries C. difficile assay has performance and workflow characteristics comparable to those of other established molecular CDI diagnostic tests. The Aries assay also provides wider flexibility with a unique mixture of batched and “sample-to-answer” testing. It can be concluded that for the diagnosis of CDI, NAATs, such as the Aries, Xpert, and BD Max assays, can provide highly sensitive and specific results in a considerably shorter time than with other available nonmolecular methods. However, future studies evaluating the different diagnostic algorithms involving NAATs are required to understand the complete potential of these assays in CDI diagnosis.
